# Evaluation of the sentinel surveillance system for influenza-like illnesses in the Greater Accra region, Ghana, 2018

**DOI:** 10.1371/journal.pone.0213627

**Published:** 2019-03-14

**Authors:** Francis Sena Nuvey, Elijah Paa Edu-Quansah, George Khumalo Kuma, John Eleeza, Ernest Kenu, Samuel Sackey, Donne Ameme, Mahamat Fayiz Abakar, Katharina Kreppel, Richard Bongo Ngandolo, Edwin Afari, Bassirou Bonfoh

**Affiliations:** 1 Ghana Field Epidemiology and Laboratory Training Program, Department of Epidemiology and Disease Control, School of Public Health, University of Ghana, Accra, Ghana; 2 Ghana Health Service, Ministry of Health, Accra, Ghana; 3 Institut de Recherche en Élevage pour le Développement, N'Djamena, Chad; 4 Nelson Mandela African Institute of Science and Technology, Arusha, Tanzania; 5 Centre Suisse de Recherches Scientifiques en Côte d’Ivoire, Abidjan, Côte d'Ivoire; 6 Swiss Tropical and Public Health Institute, Basel, Switzerland; 7 University of Basel, Basel, Switzerland; George Mason University, UNITED STATES

## Abstract

**Background:**

Influenza-like Illness (ILI) is a medical diagnosis of possible influenza or another respiratory illness with a common set of symptoms. The deaths of four schoolchildren, during a pandemic influenza outbreak in December 2017 in Ghana, raised doubts about the ILI surveillance system’s performance. We evaluated the ILI surveillance system in the Greater Accra region, Ghana, to assess the system’s attributes and its performance on set objectives.

**Methods:**

CDC guidelines were used to evaluate the data of the ILI surveillance system between 2013 and 2017. We interviewed the surveillance personnel on the system’s description and operation. Additionally, routinely entered ILI data from the National Influenza Center provided by the six sentinel sites in Accra was extracted. We sampled and reviewed 120 ILI case-investigation forms from these sites. Surveillance activities were examined on system’s performance indicators, each being scored on a scale of 1 to 3 (poorest to best performance).

**Results:**

All population and age groups were under ILI surveillance over the period evaluated. Overall, 2948 suspected case-patients, including 392 (13.3%) children under-five were reported, with 219 being positive for influenza virus (Predictive value positive = 7.4%). The predominant influenza subtype was H3N2, recorded in 90 (41.1%) of positive case-patients. The system only met two out of its four objectives. None of the six sentinel sites consistently met their annual 260 suspected case-detection quota. Samples reached the laboratory on average 48 hours after collection and results were disseminated within 7 days. Of 120 case-investigation forms sampled, 91 (76.3%) were completely filled in.

**Conclusions:**

The ILI surveillance system in the Greater Accra region is only partially meeting its objectives. While it is found to be sensitive, representative and timely, the data quality was sub-optimal. We recommend the determination of thresholds for alert and outbreak detection and ensuring that sentinel sites meet their weekly case-detection targets.

## Introduction

Influenza-like illnesses (ILI), often also called acute respiratory infection or flu-like syndrome, are acute viral infections of the respiratory tract with similar signs and symptoms to influenza. ILI is a syndrome and affected persons may become infectious before, during or after the onset of symptoms. The pathogen can be transmitted both directly (by droplets) and indirectly through contact with contaminated fomites. Children, the elderly and pregnant women, as well as persons with chronic illnesses or immunosuppression are at the highest risk for morbidity and mortality from ILIs [1,2].

According to the World Health Organization (WHO), most influenzas in the global circulation are of zoonotic origin. Sub-types implicated in epidemics include H1N1, H5N1, H7N9, H7N7 and H3N2 [3]. Influenza has a global annual attack rate of 5–10% in adults and 20–30% in children, causing between 3–5 million cases of severe illness and about 500,000 deaths yearly [4]. Pandemics of influenza have had high fatality rates in the past and robust surveillance systems are key to global efforts to prevent similar outbreaks [5]. Due to the epidemic-prone nature of influenza pathogens and their high propensity for mutations, the World Health Organization (WHO) recommends strict adherence to infection control and prevention measures including increased handwashing during peak flu seasons [2].

In Ghana, laboratory-based surveillance for Respiratory Tract Infections (RTIs) only tests for influenza in suspected cases. RTIs remain a major cause of morbidity and mortality in Ghana ranking second among the top 10 diseases seen at outpatient departments (OPDs) in healthcare facilities across the country, in 2016 [6]. Respiratory diseases may occur because of an invasion of a susceptible host by microbes including bacteria and viruses. Three main types of influenza viruses exist, namely; influenza A, B, and C but epidemics are often linked to the influenza A strain [7]. Influenza surveillance data between 2012 and 2014 in Ghana indicated 1041 positive influenza cases out of a total 8601 respiratory samples tested, with 6 different subtypes [influenza A (H3, H1N1, H1, H5) and influenza B (Victoria, Yamagata)] identified [8].

Ghana began influenza surveillance in 2007 to obtain data on strains in circulation. The National Influenza Center (NIC) was formally recognized by the WHO in 2010 after the 2009 pandemic influenza [9]. The NIC is a member of the WHO Global Influenza Surveillance and Response System (GISRS) and is located in the Department of Virology of the Noguchi Memorial Institute of Medical Research (NMIMR). In collaboration with the Ghana Health Service (GHS) and the Ministry of Defense (MOD), it currently operates sentinel surveillance for influenza in 27 sites across all regions in Ghana with support from the U.S. Naval Medical Research Unit No. 3 (NAMRU-3), Centers for Disease Control and Prevention (CDC) and WHO [8]. ILI surveillance is conducted all year round across the sentinel sites.

The ILI surveillance system aims to detect early unusual events indicating a change in circulating influenza sub-types, identify and monitor vulnerable groups for influenza, determine influenza thresholds and detect antiviral resistance. In December 2017, an outbreak of influenza in a school in the Ashanti region of Ghana was followed by the death of four children (Case Fatality Rate = 5.2%) [10]. This raised concerns about the effectiveness of the influenza surveillance system, particularly that in the Greater Accra region of Ghana, which recorded no alerts over the past five years. We evaluated the effectiveness of the ILI sentinel surveillance system to determine if its objectives are being met and to assess its attributes and usefulness. The findings of this study are important in order to give useful recommendations to improve the current system.

## Materials and methods

We described the attributes and effectiveness of the ILI sentinel surveillance system in the Greater Accra region (GAR) of Ghana using a descriptive cross-sectional survey. We extracted and evaluated routinely recorded ILI data between January 2013 and December 2017.

### Study area

**Fig 1:**
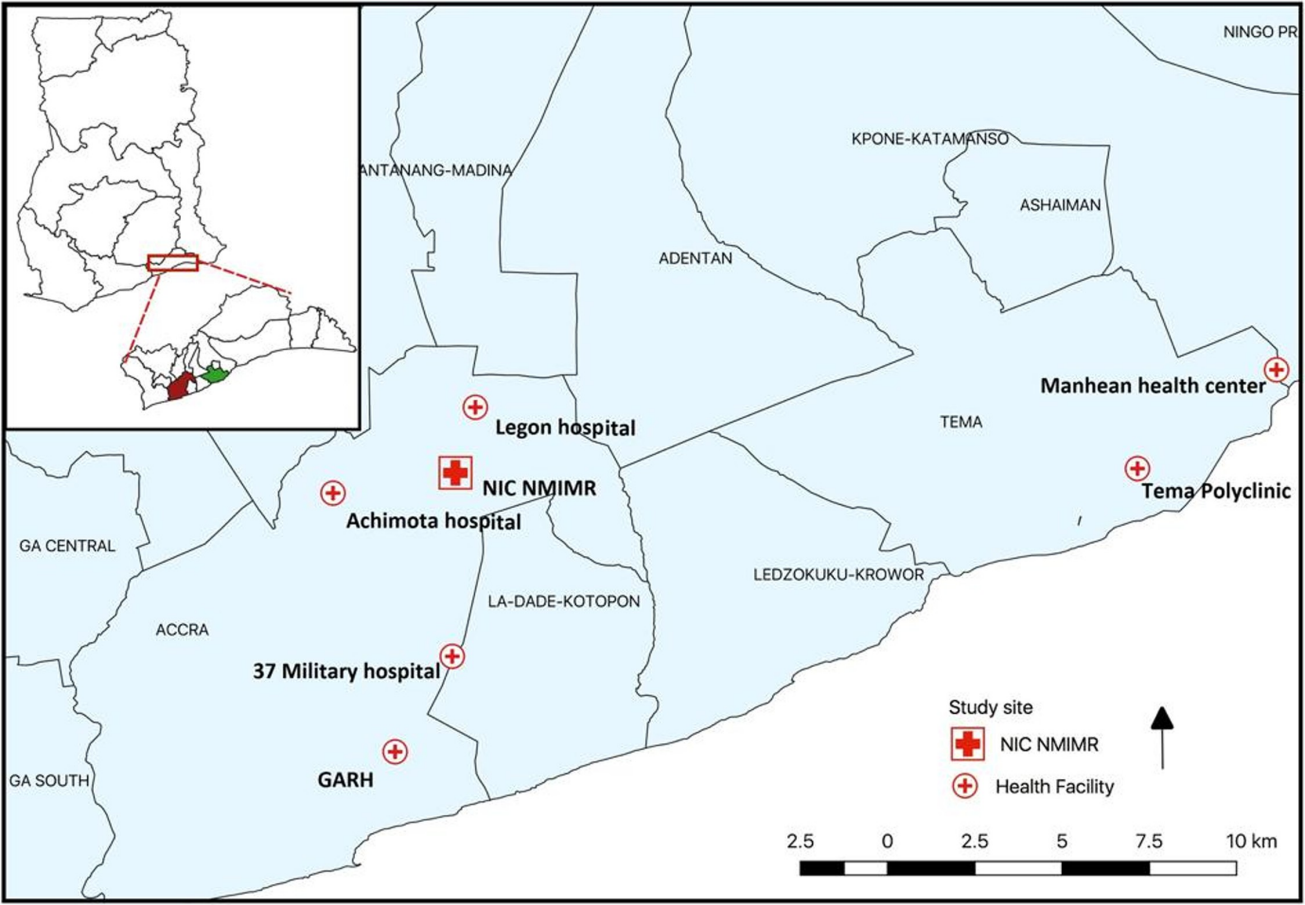
Map of the Greater Accra region showing sentinel facilities and National Influenza Center.

The GAR is one of ten regional demarcations in Ghana with a population of about 5 million people [11]. As the most densely populated region in Ghana, it is mainly an urban settlement and lies in the southeastern part of the country along the coast of the gulf of Guinea. It is the administrative capital of Ghana with over 500 public and private health care facilities. Six of these facilities in the region conduct ILI surveillance including four GHS facilities, one military and one quasi-government facility (see Fig 1). They fall under the three main levels of healthcare delivery in Ghana; primary, secondary and tertiary. The Manhean health center provides primary health services, while secondary healthcare providers involved in ILI surveillance are Tema Polyclinic, Achimota hospital and University of Ghana Hospital, Legon. Lastly, the Greater Accra Regional Hospital (GARH) and 37 Military hospital provide tertiary care.

### Data collection procedure

Data on ILI from all six sentinel facilities in GAR was extracted and abstracted from the NIC database into Microsoft Office Excel format. Additionally, we selected three sentinel facilities, based on the different levels of care they provided, and obtained permission for site visits. These sentinel sites; GARH, Achimota hospital and Manhean Health Center, were visited for at least one week each and their records were collected and reviewed. The researchers interviewed all personnel directly involved in ILI surveillance and also partook in surveillance activities while observing practices.

The staff members were interviewed using a structured questionnaire and guided interviews on their knowledge on ILI surveillance activities. Forty case investigation forms were randomly sampled from each of the three facilities, using Microsoft Excel. The unique serial numbers on case investigation forms for each sentinel site were used to retrieve the randomized forms for examination. In addition, we collected and reviewed ILI registers at the sites.

### Data analysis

The evaluation was conducted using the CDCs guidelines for evaluating public health surveillance systems [12]. Nine attributes: simplicity, flexibility, data quality, acceptability, sensitivity, predictive value positive, representativeness, timeliness and stability, usefulness and the utility of the system to achieve its objectives were evaluated. The indicators for each attribute or characteristic measured were scored one point each. Based on the evaluator’s assessment, an indicator is scored zero (0) if the key finding from evaluation do not support the indicator assessed in relation to the attribute. A score of one (1) is given to each indicator that supports the attribute assessed. The assessment scores were then summed and divided by the total number of indicators used in evaluating each attribute. Attributes with relative scores of more than two-thirds of the total score, were considered as major strengths of the system and scored 3 overall. Those with less than one-third were major weaknesses (Overall score = 1). Scores between one-third and two-thirds were relative strengths (Overall score = 2). Scores were on a scale of 1 to 3: poorest to best performance, respectively.

### Ethics statement

The evaluation was done within the framework of Integrated Disease Surveillance and Response matrix implemented by the Ghana Health Service and therefore did not have to receive formal review by Ethical Review Committees. The Field Epidemiology and Laboratory Training Programme and National Influenza Center in Ghana approved the study. Permission was sought and obtained from the Public Health Directorate of Ghana Health Service and authorities in the sentinel facilities before commencement of the evaluation. All respondents provided informed, written consent and were assured of confidentiality.

## Results

### Operation of the ILI sentinel surveillance system

The surveillance system was found to be utilizing the syndromic approach by screening suspected cases and thereafter conducting further laboratory confirmatory tests on collected nasopharyngeal or oropharyngeal specimen. A Reverse Transcriptase-Polymerase Chain Reaction (rRT-PCR) is used to confirm real influenza cases from among those suspected using the influenza case definition and determine the influenza virus sub-type.

We found that data on patients meeting the ILI case definition (S2 Table) from the sentinel sites are collected together with nasopharyngeal or oropharyngeal specimen. Specimen are stored in sample bottles with Virus Transport Media (VTM) in a cold chain system and submitted to NMIMR for laboratory confirmation. Each site is required to suspect five cases weekly using the case definition. Thus, the expected annual total for each site is 260 suspected cases.

Firstly, clinicians at the OPDs suspect cases and trained personnel (i.e. nurses, laboratory scientists and surveillance officers) collect the specimen for storage in designated refrigerators at the Disease Control Units. Case detection is, in most cases, directly done by ILI surveillance team members at the OPDs. Socio-demographic, epidemiologic and clinical data are collected on each case using case investigation forms.

The NIC supplies the sentinel sites with the tools including case investigation forms, VTM, and specimen bottles, for the system’s operation. They contact the sentinel sites via phone calls and visit the sentinel sites in the GAR at least twice every week to pick up specimen. The NIC reports results from the polymerase chain reaction laboratory test to each sentinel site, the National Surveillance Department (NSD), and WHO via e-mail. Data is entered into the FluNet system, a WHO (GISRS) global database specifically designed for influenza surveillance, weekly. Sentinel sites receive printed laboratory results from the NIC averagely every 7 days.

**Fig 2:**
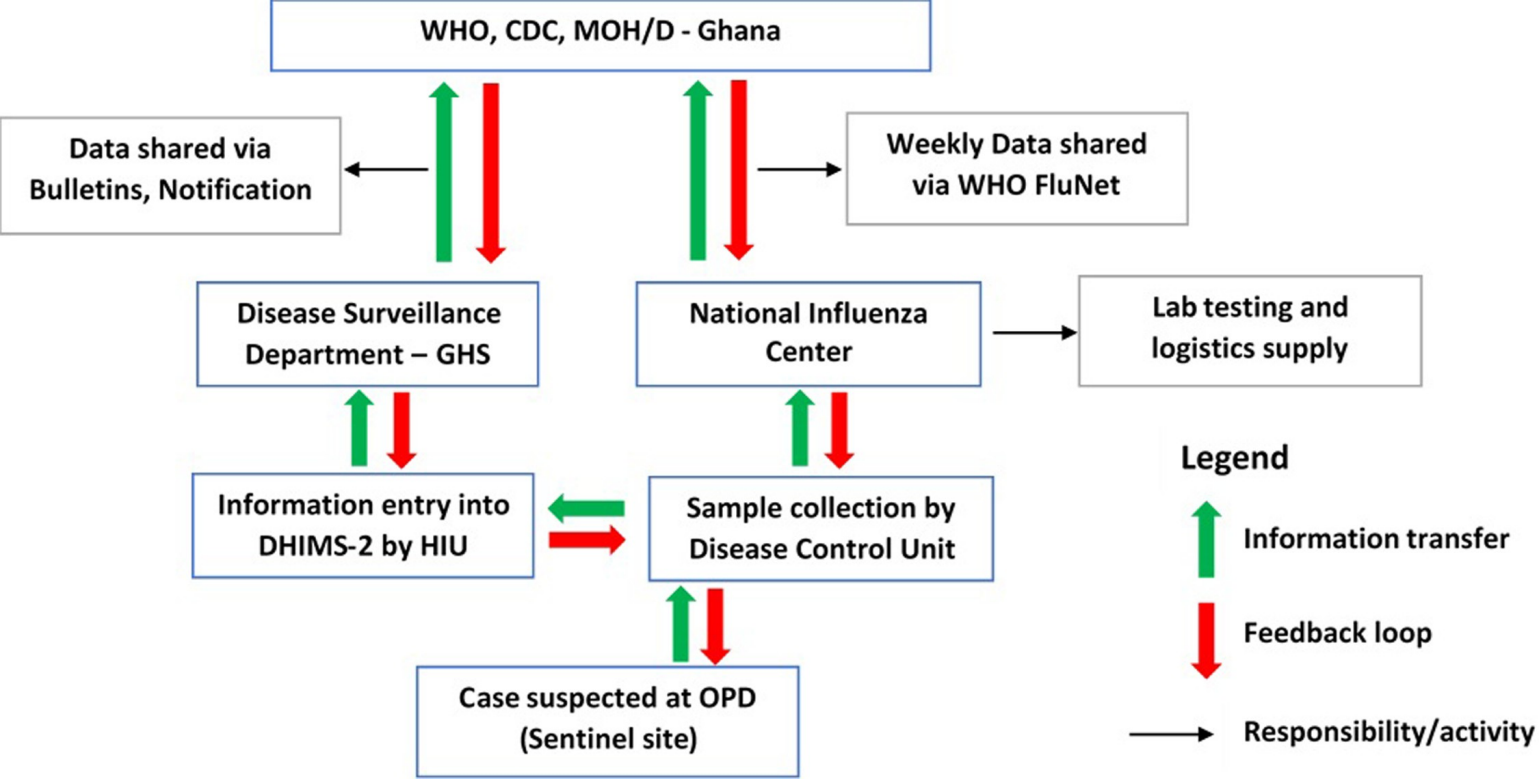
Direction of information and data flow in the ILI sentinel surveillance system, Greater Accra region, Ghana, 2013–2017.

At the facility level, health information units (HIU) enter the data into the District Health Information Management System 2 (DHIMS 2). ILI data entry into DHIMS 2 started in 2017. The GHS, NIC and other stakeholders periodically hold review meetings to discuss influenza activities and publish the data generated by the system in reports and journals. The information and data flow in the ILI surveillance system is further illustrated in Fig 2.

### Influenza detection

Of the 2948 suspected cases tested, 219 (7.4%) cases were tested positive for influenza viruses. The predominant strain detected over the period was influenza A (157/219); A (H3N2) = 90 and A (H1N1) pdm09 = 62. Influenza B virus (62/219) lineages identified include B (Victoria) = 30 and B (Yamagata) = 17. The remaining 20 confirmed specimen were not sub-typed; A = 5 and B = 15 (see S1 Table).

### Characteristics and attributes of the ILI surveillance system

#### Utility of the ILI sentinel surveillance system in attaining set objectives

The ILI surveillance system has four objectives: early detect events that show change in severity or patterns of influenza infection or emergence of new strains, determine influenza thresholds, identify and monitor at risk groups and detect antiviral resistance in circulating strains. The system met two out of its four objectives over the period evaluated. It was able to detect and characterize influenza viruses in circulation, as from influenza A and B lineages, and established the groups of persons most at risk of influenza infection. It however did not set thresholds, which is the minimum number of suspected cases above which the system is alerted, and was not performing antiviral resistance testing.

#### Surveillance system attributes

**Usefulness:** Data generated by the system informed the choice of vaccines used in controlling influenza outbreaks in Ghana. In addition, WHO and CDC use the system’s information to monitor influenza activity globally, through sharing of confirmed influenza samples. Tables 1 and 2 show the key findings on the indicators and scores for all the qualitative and quantitative attributes evaluated respectively.

**Table 1:** Influenza-like illnesses surveillance system qualitative attribute indicators evaluated, key findings, assessment scores (0 = key finding does not support the attribute and 1 = key finding supports the attribute) and overall scores (1 to 3), Greater Accra region, 2013–2017.

Attribute, issue	Indicators evaluated	Key findings	Assessment score	Overall Score
**Simplicity**	• Amount of data obtained from each suspected case • Number of reporting sources • Surveillance staff perception of case definition’s simplicity • Training needed to conduct ILI surveillance and testing • Any laboratory testing of specimen at sentinel sites • Nature of specimen collection • Follow-up period for confirmed cases	• Demographic, clinical and epidemiological data • Reporting by only OPDs • Simple case definition • Specialized training required • No testing done at sites • Nasal or throat swabs • Confirmed cases followed up for 5 days	0 1 1 0 0 0 1	**1**
**Flexibility**	• Modification of case definition at facility level • Use of case definition in detecting other health events	• Symptoms added to those in IDSR case definition • Used in surveillance of SARI	1 1	**3**
**Stability**	• Proportion of evaluated years during which all sites were detecting cases • Frequency of interruption in system’s operation • Number of unscheduled outages of system’s computer • Funding source for ILI surveillance • Difference between desired and observed time with data flow	• 3/5 years (60%) • None • None • Mainly donor funded • Time periods conform with WHO standards	0 1 1 0 1	**2**
**Utility of system in meeting of surveillance objectives**	• Number of alerts detected, classifying of influenza strains • Establishment of thresholds • Identification of vulnerable groups • Detection of resistance to influenza antivirals	• No alerts, viruses are sub-typed • Not done • Met • Resistance not tested	1 0 1 0	**2**

**Table 2:** Influenza-like illnesses surveillance system quantitative attribute indicators evaluated, key findings, assessment scores (0 = key finding does not support the attribute and 1 = key finding supports the attribute) and overall scores (1 to 3), Greater Accra region, 2013–2017.

Attribute, issue	Indicators evaluated	Key findings	Assessment score	Overall Score
**Acceptability**	• Proportion of facilities reporting per surveillance period • Percentage of completed case report forms • Proportion of sentinel facilities meeting case detection quota • Mean time from case detection to lab testing • Proportion of surveillance staff satisfied with feedback from NIC	• 5/6 (83%) • 76% (91/260) • 1/4 (25%) • 2.0 ± 0.7 days • 10/10 (100%)	1 0 0 1 1	**2**
**Timeliness**	• Mean time between onset of symptoms and case detection • Mean time between sample collection and laboratory testing • Proportion of sentinel sites that confirm receipt of test results (feedback) within 7 days	• 10.2 ± 1.9 days • 2.0 ± 0.7 days • 4/4	1 1 1	**3**
**Representativeness**	• Variability in age distribution of cases • Variability in sex distribution of cases	• Median age was 30 years (range: 3 weeks–90 years) • Females were 60% (1775/2948)	1 1	**3**
**Predictive value positive**	• Proportion of reported cases testing positive	• 219/2948 (7.4%)		
**Data quality**	• Percentage of completed case report forms • Variability in data at different levels • Completeness of ILI registers	• 76% (91/120) • Mean number of cases at sites (125.6) ≈ Mean number of cases at NIC (124.4) • Results not entered in registers at site	0 1 0	**2**

**Sensitivity:** The ILI system in GAR has been able to confirm influenza cases in each of the years evaluated (S1 Table). Evidence of aggregate data from Ghana can also be found in the WHO FluNet system, available free at http://apps.who.int/flumart/Default?ReportNo=1.

**Predictive value positive (PVP):** The predictive power of the ILI case definition is tested using rRT-PCR. Overall PVP for the period was 7.4% (range 4.7%– 14.8%). See S3 Table for yearly PVPs over the evaluated period.

**Simplicity:** Surveillance personnel interviewed identified case definition to be simple. Only OPDs partake in ILI surveillance and follow-up of confirmed cases done within 5 days. However, specialized training is required for specimen collection and laboratory confirmation, data collected on each suspected case is comprehensive and laboratory testing is not done at sentinel sites.

**Flexibility:** Modification of the ILI system in 2016, enabled detection of Severe Acute Respiratory Infections (SARI) with no difficulty. Furthermore, addition of other common respiratory symptoms to case definition at the sites did not disrupt the system.

**Data quality:** Key surveillance information was completely provided in 76% (91/120) of case investigation forms sampled and data extracts were comparable at sentinel facilities and the NIC. In spite of this, only two sites: GARH and Achimota hospital, had 2017 influenza data entered in the DHIMS-2 platform and laboratory results were not entered in ILI registers at the three sites.

**Fig 3:**
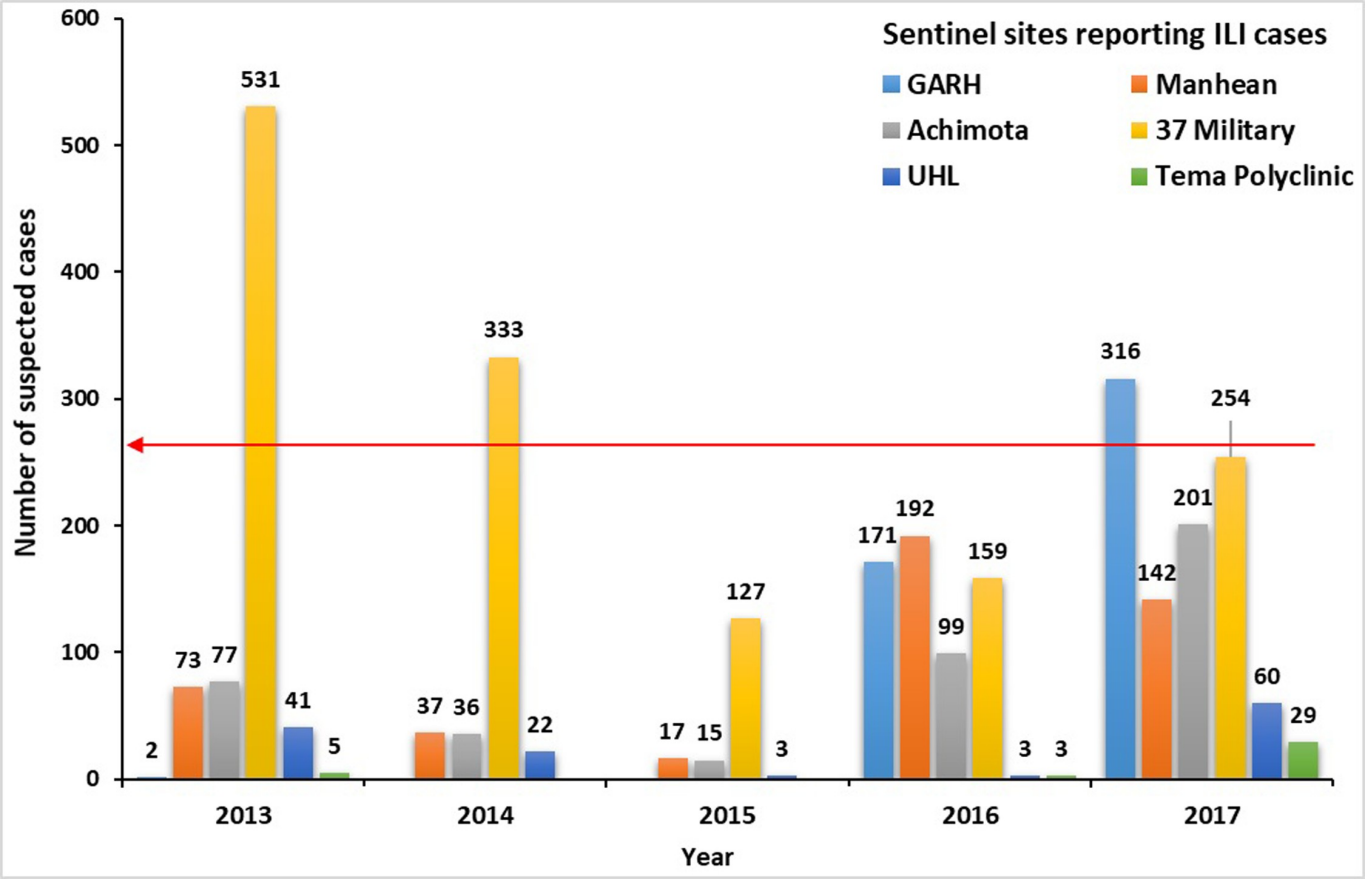
Expected and total number of suspected Influenza-like illnesses cases per sentinel site from 2013 to 2017 in the Greater Accra region in Ghana.

**Acceptability:** On average, five out of the six sentinel sites detect influenza cases each year (83%) and about 80% of case investigation forms sampled were completely filled out by ILI personnel. All sentinel staff interviewed reported satisfaction with feedback from NIC and test results from collected specimen are on average tested within 2 days. Most of the sites (5/6) however, do not meet the case detection quota for each year with some recording as low as three suspected cases per year only (see Fig 3).

**Representativeness:** Age and sex distribution of total cases detected reflect the general distribution of Ghana’s population. The median age of cases was 30 years (range: 3 weeks to 90 years). Females constituted 60% (1775/2948) of cases.

**Timeliness:** It takes on average 10 days between symptom onset and detection at facilities. The majority of specimens are tested within 48 hours after collection and results are disseminated within 7 days. This conforms with the WHO set standard timelines for influenza surveillance.

**Stability:** Data flow in the system conforms to set standards; testing is routinely done within 48 hours after case detection and results are released within 7 days to the sites on average. The system is sustainable with donors mainly funding its operation. There was not a time during the period evaluated when no site recorded cases, but all sites detected cases only for 60% of the period.

## Discussion

Influenza causes considerable morbidity and about 500,000 deaths per year globally. The disease is highly infectious with high pandemic potential. Therefore, worldwide surveillance systems to record Influenza-like illnesses (ILI) in real time and detect possible influenza outbreaks are essential to prevent and control epidemics. Ghana’s ILI surveillance system since its inception in 2007, aims to early detect changes in circulating influenza, identify vulnerable groups to influenza infection, determine influenza thresholds and detect antiviral resistance to influenza viruses. The late detection of a pandemic influenza outbreak in Ghana raised doubts about the ILI surveillance system’s performance.

Our study provides evidence, that the ILI sentinel surveillance system in the Greater Accra Region (GAR), Ghana, is only partially meeting its objectives because it did not have thresholds for alerting the health system and does not perform antiviral resistance testing. It is sensitive and timely in detecting influenza cases but of low predictive value positive (PVP). It is representative of the population under surveillance and flexible to modifications. The system is fairly stable and acceptable to key stakeholders; the quality of data is relatively high. Predominant influenza subtype in circulation is influenza A (H3N2) virus. The sentinel sites consistently failed to meet their case detection quotas annually over the period evaluated. Despite these apparent weaknesses in the ILI system, the good performance of the laboratory component is commendable and key to detection of novel influenza viruses for prompt response. Even though the WHO’s standards for influenza surveillance alluded to the possibility of resource limitation hindering achievement of all influenza surveillance system objectives, it advocates for influenza surveillance systems capable of collecting the minimum amount of data needed for decision making [5].

For the past two decades of influenza surveillance in Ghana, the system proved its utility by its ability to detect and classify circulating strains as well as providing key information for public health action. Similar findings were made in the South African [13] and Madagascar [14] influenza surveillance systems. Even though the PVP is low, it generally conforms to other syndromic surveillance systems with broad case definitions but specific for respiratory diseases in this case, aimed at maximizing influenza case detection [15].

Nevertheless, the unmet objectives, lack of thresholds and antiviral resistance testing, of the system require attention. The lack of antiviral resistance testing to detect the emergence of treatment resistant strains and the absence of thresholds preventing the issuing of alerts, are a major drawback. This situation is not specific to the GAR alone but is found in all sites in Ghana. In addition, the mainly conservative management of clinical signs and symptoms, of confirmed influenza cases in the health system, without the use of recommended antiviral agents, may partly explain the absence of antiviral susceptibility testing. These shortfalls may, however, be caused by a lack of capacity or resources (antiviral drugs and laboratory reagents) at the sentinel facilities to determine thresholds for influenza alerts and test for antiviral resistance, as was observed in evaluations done in other settings [16,17].

Threshold establishment for disease surveillance is paramount to be able to alert the health system early when outbreaks occur for prompt public health action [1]. A study to test the Moving Epidemic Method in determining thresholds for ILI and SARI surveillance systems in Europe, was able to detect epidemic periods with few or no false alarms in different countries [18]. Other methods employed in Cambodia [19] and Australia [20] had similar findings. The NIC must take the lead using the WHO manual [21], to choose an epidemic threshold determination method and train focal persons in the facilities to use it, to ensure this key information is available.

Even though implementing an integrated (human and animal) surveillance could be cost-effective and improve case detection and response [22], discussions with ILI surveillance personnel at sentinel sites revealed the absence of a link between influenza surveillance in humans and animals. Human infection with zoonotic influenza strains is possible and may cause mild to severe form of the disease. There have been recorded episodes of pandemics in humans over a century due to cross-species transmission of zoonotic respiratory viruses including influenza viruses, notably Spanish flu (1918), severe acute respiratory syndrome (2003) and swine flu (2009), resulting in high morbidity and mortality globally [23,24]. Thus, the importance of a One-Health approach in the surveillance and response with control of pandemics in an increasingly globalized world is evident. Research findings in Africa, Europe and the Americas, have shown incremental gains derived when interventions are integrated between human and animal health systems [25]. The NIC and Veterinary Service Department in Ghana should collaborate to formalize protocols for engaging each other to integrate influenza surveillance systems in humans and animals, using a One Health approach, as this can provide additional key information on possible cross-species transmission in the country and enhance savings. The usage of one laboratory to test for both human and animal pathogens in Canada was shown to cost about 30% less than the combined original operational costs of testing individually in both laboratories [26].

In Ghana, the timeliness of the system in case detection, laboratory confirmation and result dissemination is commendable. This strength may be as result of the weekly visits NIC makes to sentinel sites to collect specimen, supply virus transport media in specimen bottles and transfer results. The NIC should further take advantage of these visits and collaborative meetings to ensure each site meets weekly case detection quotas. Focal persons at the various sites must take the lead.

## Conclusion

In spite of the usefulness and fair performance of ILI surveillance in GAR on indicators evaluated, it is only partially meeting its set objectives. It is sensitive in detecting circulating influenza types, representative of the population under surveillance and timely. However, sentinel sites do not consistently meet annual case detection quotas. There is the need to address shortfalls in the system’s objectives as well as improving case detection at sentinel facilities. This would ensure that the successes chocked are not undermined, thereby preventing increasing morbidity and mortality related to influenza infections. Failure of the system to address these shortfalls would also affect Ghana’s contribution to the WHO Global Influenza Surveillance and Response System. Considering the zoonotic character of most influenza viruses, it is important that a One Health approach is adopted with influenza surveillance in Ghana. We propose strict adherence to case detection targets by individual sentinel sites and determination of alert thresholds for the system to allow for a more effective monitoring of influenza activity in the region for prompt public health actions.

## Supporting information

S1 Table Influenza cases detected by subtype from ILI sentinel surveillance (2013–2017), Greater Accra region, Ghana. (DOCX)

S2 Table ILI case definitions used for screening and enrolment by the ILI sentinel surveillance system, Greater Accra region, Ghana, 2013–2017. (DOCX)

S3 Table Predictive value positives (PVP) of ILI surveillance in Greater Accra region, Ghana, 2013–2017. (DOCX)

S1 File CDC Checklist for assessment of health surveillance systems. (DOCX)
