# Supplementary material for: Evaluation of the sentinel surveillance system for influenza-like illnesses in the Greater Accra region, Ghana, 2018
Source: PLoS One. 2019 Mar 14;14(3):e0213627. doi: 10.1371/journal.pone.0213627 (PMC6417674; doi:10.1371/journal.pone.0213627)
Supplement: S1 Table — (DOCX) [file pone.0213627.s001.docx]

**Table S1. Influenza cases detected by subtype from ILI sentinel surveillance (2013–2017), Greater Accra region, Ghana**

|  | **Influenza type** | | | | | |  |
| --- | --- | --- | --- | --- | --- | --- | --- |
| **Year** | **A(H1N1)pdm09** | **A(H3N2)** | **B(Victoria)** | **B(Yamagata)** | **A(not subtyped)** | **B(not subtyped)** | **Total** |
| 2013 | 2 | 17 | - | - | - | 15 | 34 |
| 2014 | 8 | 16 | 13 | - | - | - | 37 |
| 2015 | 7 | 9 | - | 8 | - | - | 24 |
| 2016 | 25 | 23 | 16 | 7 | 5 | - | 76 |
| 2017 | 20 | 25 | 1 | 2 | - | - | 48 |
| **Total** | **62** | **90** | **30** | **17** | **5** | **15** | **219** |
