# Supplementary material for: Evaluation of the sentinel surveillance system for influenza-like illnesses in the Greater Accra region, Ghana, 2018
Source: PLoS One. 2019 Mar 14;14(3):e0213627. doi: 10.1371/journal.pone.0213627 (PMC6417674; doi:10.1371/journal.pone.0213627)
Supplement: S2 Table — (DOCX) [file pone.0213627.s002.docx]

**Table S2. ILI case definitions used for screening and enrolment by the ILI sentinel surveillance system, Greater Accra region, Ghana, 2013–2017.**

| Suspected case definition | Any person with sudden onset of fever (history/measured) of ≥38℃ (axillary) and cough and/ or other respiratory signs with onset within the last 10 days |
| --- | --- |
| Confirmed case definition | A case that meets the clinical case definition and has a positive laboratory result for influenza virus |
