# Supplementary material for: Evaluation of the sentinel surveillance system for influenza-like illnesses in the Greater Accra region, Ghana, 2018
Source: PLoS One. 2019 Mar 14;14(3):e0213627. doi: 10.1371/journal.pone.0213627 (PMC6417674; doi:10.1371/journal.pone.0213627)
Supplement: S3 Table — (DOCX) [file pone.0213627.s003.docx]

**Table S3. Predictive value positives (PVP) of ILI surveillance in Greater Accra region, Ghana, 2013–2017**

| **Year** | **Total suspected cases** | **Total positives** | **PVP (%)** |
| --- | --- | --- | --- |
| 2013 | 729 | 34 | 4.66 |
| 2014 | 428 | 37 | 8.64 |
| 2015 | 162 | 24 | 14.82 |
| 2016 | 627 | 76 | 12.12 |
| 2017 | 1002 | 48 | 4.79 |
| **Total** | 2948 | 219 | 7.43 |
