# Supplementary material for: Evaluation of the sentinel surveillance system for influenza-like illnesses in the Greater Accra region, Ghana, 2018
Source: PLoS One. 2019 Mar 14;14(3):e0213627. doi: 10.1371/journal.pone.0213627 (PMC6417674; doi:10.1371/journal.pone.0213627)
Supplement: S1 File — (DOCX) [file pone.0213627.s004.docx]

**Public Health importance measure**

- Prevalence/mortality rates, QALY, DALY
- Preventability
- Costs associated with treatment

**Purpose and Operation of Surveillance System**

- Objectives of surveillance system
- Planned and actual uses of data generated
- Case definition
- Location of surveillance system
- Components of surveillance system
  - Population under surveillance
  - Period of time data collected
  - What data are collected and how they are collected
  - Reporting sources of data
  - How is Data analysed and disseminated
  - Policy for data confidentiality
  - Storage of data generated
- Flow chart of information flow

**Resources used to operate the system**

- Funding source: example Collaboration between state and NGO
- Personnel
- Equipment used
- Other resources

**Usefulness of system**

- Actions taken from analysis and interpretation of data
- Meeting objectives
- Detection of diseases in timely manner
- Provides estimates of magnitude of morbidity and mortality related to disease
- Detects trends in occurrence
- Detecting epidemics
- Other anticipated uses of data generated

**Simplicity**

- Flow chart of flow of data (number of reporting sources / Level of integration with other systems )
- Simplicity of case definition
- Amount of data collected on each case
- Method of data collection
- Amount of follow up period
- Method of data analysis
- Laboratory requirements (staff, complexity)

**Flexibility**

- Retrospective analysis of how system responded to new case definition
- Use of case definition in detection of other health events

**Data quality**

- Quality of data (Similarity of data at different levels)
- Completeness of data

**Acceptability**

- Facility participation rate
- Completeness of report forms
- Timeliness of reporting
- Feedback availability
- Timeliness of meeting set targets

**Sensitivity**

*Qualitative*

- Ability to detect cases and/or outbreaks

*Quantitative*

- Proportion of total (real) cases in the population detected by surveillance system

**Predictive value positive (PVP)**

- Proportion of suspected cases confirmed by laboratory testing

**Representativeness**

- Characteristics of population under surveillance. Example Age, occupation or sex distribution of cases
- Departmental/facility participation

**Timeliness**

- Average time between symptom onset and reporting to hospital
- Time between collection of samples and submission to laboratory for testing
- Time between collection of samples by laboratory and provision of test result to reporting facility

**Stability**

- Number of unscheduled downtimes of surveillance system (system, equipment e.g. Computers, etc.)
- Proportion of time system is operating fully
- Availability of regular funding for system’s operation
- Difference between desired and observed time with data flow
